# Antimalarial Drug Repurposing of Epirubicin and Pelitinib in Combination with Artemether and Lumefantrine

**DOI:** 10.3390/ph18070956

**Published:** 2025-06-25

**Authors:** Douglas O. Ochora, Reagan M. Mogire, Bernard M. Murithi, Farid Abdi, Erick N. Ondari, Rael J. Masai, Edwin Mwakio, Agnes Cheruyiot, Abiy Yenesew, Hoseah M. Akala

**Affiliations:** 1 Department of Biological Sciences, School of Pure and Applied Sciences, Kisii University, Kisii P.O. Box 408-40200, Kenya; dreondari@kisiiuniversity.ac.ke (E.N.O.); jjepkogei@gmail.com (R.J.M.); 2 Center for Research on Genomics and Global Health, National Human Genome Research Institute, National Institutes of Health, Bethesda, MD 20894, USA; reaganmoseti@gmail.com; 3 Medical Biotechnology and Immunotherapy Research Unit, Department of Integrative Biomedical Sciences, Institute of Infectious Disease and Molecular Medicine (IDM), Faculty of Health Sciences, University of Cape Town (UCT), Private Bag X3, Cape Town 7701, South Africa; murithibenn@gmail.com; 4 Kenya Medical Research Institute (KEMRI), Walter Reed Army Institute of Research-Africa, Kisumu P.O. Box 54-40100, Kenya; faridsalim2030@gmail.com (F.A.); edwin.mwakio@usamru-k.org (E.M.); agnes.cheruiyot@usamru-k.org (A.C.); hoseaakala@yahoo.com (H.M.A.); 5 Department of Chemistry, Faculty of Science and Technology, University of Nairobi, Nairobi P.O. Box 30197-00100, Kenya; ayenesew@uonbi.ac.ke

**Keywords:** drug repurposing, epirubicin, pelitinib, drug combination, artemether, lumefantrine, malaria, *Plasmodium falciparum*

## Abstract

**Background:** Drug therapy remains the principal management strategy for malaria but is increasingly challenged by the emergence of drug-resistant malaria parasites. The need for new antimalarial drugs is urgent, yet drug discovery and development are hindered by high costs, long durations, and safety concerns that prevent approval. The current study aimed to determine antiplasmodial activities of approved drugs in combination with artemether (ART) and lumefantrine (LU). **Methods**: Using the SYBR Green I assay test, this study investigated the efficacy of epirubicin (EPI) and pelitinib (PEL) combined with ART and LU at fixed drug–drug ratios (4:1, 3:1, 1:1, 1:2, 1:3 and 1:4) and volume/volume. These combinations, as well as single drug treatments, were tested against cultured strains of *Plasmodium falciparum* (W2, DD2, D6, 3D7 and F32-ART) and fresh and cultured clinical isolates. The fifty percent inhibition concentration (IC_50_) and a mean sum of fifty percent fractional inhibition concentration (FIC_50_) were determined. **Results**: Synergism was observed when EPI was combined with both ART and LU across all fixed ratios with a mean of mean FIC_50_ values of <0.6. The combination of LU and EPI against the 3D7 strain demonstrated the highest efficacy with a synergism FIC_50_ value of 0.18. Most combinations of PEL with ART and LU showed antagonism (FIC_50_ > 1) when tested against strains of *P. falciparum* and clinical isolates. **Conclusions**: This study underscores the utility of alternative drug discovery and development strategies to bypass cost, time, and safety barriers, thereby enriching the antimalarial drug pipeline and accelerating the transition from lab to market.

## 1. Introduction

Malaria remains a critical global health burden. The disease is caused by six *Plasmodium* species in humans—*Plasmodium falciparum*, *P. vivax*, *P. malariae*, *P. knowlesi*, *P. ovale curtisi* and *P. ovale wallikeri*—and is transmitted by female Anopheles mosquitoes [1]. Among these, *P. falciparum* is the most virulent, accounting for the majority of global malaria mortality [2]. In 2023, malaria was responsible for approximately 263 million clinical cases and 597,000 deaths globally, with Africa accounting for 94% of these cases and 95% of the deaths. Nigeria carried the heaviest malaria burden, with 26% of the global malaria cases in 2023 [3].

The observed malaria prevalence is mainly due to the ever-increasing epidemiological reports of multidrug-resistant strains of malaria-causing parasites to the currently used drugs [4]. To counteract antimalarial drug resistance and increase therapeutic activity, the World Health Organization (WHO) recommends the use of combination therapy as the standard treatment of malaria [5]. This has led to the current use of artemisinin-based combination therapies (ACTs) for the treatment of malaria [6]. These ACTs involve a combination of a first-acting drug, usually an artemisinin derivative, and a slow-acting partner drug. The recommended ACTs among African countries include artemether-lumefantrine (AL), dihydroartemisinin-piperaquine (DHA-PPQ), and artesunate-amodiaquine (AS-AQ) [7]. However, AL is the most widely used ACT in most African countries as the first-line treatment of uncomplicated falciparum malaria [5]. However, resistance of *P. falciparum* to currently used ACTs has been reported in various African countries [8] and in Southeast Asia [9]. This calls for alternative antimalarial therapies with novel modes of action from those that are currently used.

Antimalarial drug resistance poses a threat to the efforts towards malaria control and elimination such as early national campaigns, vector control (use of insecticidal mosquito nets and indoor residual spraying), early diagnosis and treatment [10], and the recent approval of the malaria vaccine, Mosquirix (RTS,S/AS01), by the WHO [11]. Despite all these efforts, the use of antimalarial drugs remains the main malaria management strategy [5,12]. This indicates the need for alternative, effective, and safe antimalarials. This makes the malaria situation gloomy since antimalarial drug discovery and development is slow compared with the rate of emergence of resistant malaria-causing parasites.

On this mission for alternative antimalarials, most studies have focused on plants used traditionally for the treatment of malaria as potential sources of novel antimalarial compounds [13,14,15]. Phytochemicals such as alkaloids [16], flavonoids [17], benzophenones [14,18], and xanthones [13,19] have been explored as potential antimalarial compounds. Similarly, antimalarial plant extract–extract and extract–drug combinations have been explored as alternative approaches [6,20]. These orthodox methods of drug discovery and development have proven to be slow and expensive [21]. They cost about 2.6 billion dollars [22] and take about 10–15 years [21,23]. Other drug-like compounds with high therapeutic activities have failed to obtain approval for clinical use due to safety concerns [24]. This has led to few antimalarial compounds in clinical phases of drug development [25,26] and consequently a reduced number of currently available effective antimalarial drugs [27]. For example, the development of an antiplasmodial drug candidate, DM1157 (clinical trial number NCT03490162), was halted due to safety concerns. The compound had shown antiplasmodial activity against *P. falciparum* [25]. This calls for a wider approach towards antimalarial drug discovery and development.

As a means to circumvent these challenges, current approaches towards drug discovery and development have focused on in silico approaches to accelerate drug development [28]. Advanced computer-aided drug discovery platforms and tools, machine learning [29], deep learning [30], and artificial intelligence [22] have been used for drug development.

Correspondingly, the focus on searching for antimalarial drugs is shifting towards repurposing approved drugs [31,32]. Drug repurposing involves the use of approved or investigational drugs for the treatment of a disease other than the initial indication. This strategy has been used for the discovery of antimalarial drugs such as doxycycline, tetracyclines, and clindamycin that were previously used as antibiotics [33,34]. Equally, a recent review has reported a potential repurposing of antimalarial drugs for the treatment of tuberculosis [35]. On this quest, the current study focused on antiplasmodial drug combinations of two approved drugs, pelitinib and epirubicin, that have been reported to have similar targets with *P. falciparum* proteins [36] and showed potential antiplasmodial activities in our previous study [37]. Therefore, the current study aimed to combine epirubicin and pelitinib, each with artemether and lumefantrine, providing additional possible alternatives to the current most recommended use of combination therapy in the treatment of malaria [5].

## 2. Results

For drug combinations, the mean sum FIC_50_ is grouped into three categories:

i. Synergism (FIC_50_ < 1) is where one drug potentiates the activity of another drug, leading to a greater combined effect of the two drugs than when used alone.
ii. Additivity (FIC_50_ = 1) is where the combined effect of the two drugs is the same as the effect of the individual drugs when used alone.
iii. Antagonism (FIC_50_ > 1) is where the combined effect of the two drugs is lower than the effect of the individual drugs when used alone; one drug reduces the effect of the other [6,38].

### 2.1. Antiplasmodial Activities of Artemether and Lumefantrine Each Combined with Epirubicin

**Figure 1:**
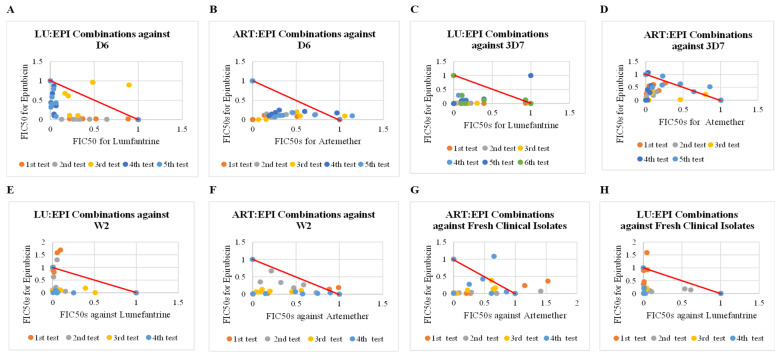
Interaction profiles of artemether and lumefantrine combinations with epirubicin against clinical isolates and cultured strains of *P. falciparum.* This figure presents a series of scatter plots illustrating the mean sum FIC_50_ values for combinations of artemether (ART) and lumefantrine (LU) with epirubicin (EPI). Each plot delineates the interactions against distinct strains of *P. falciparum* in vitro (D6, W2, 3D7) and ex vivo against clinical field isolates. Plots below the red line indicate synergism, plots along the red line indicate additivity, and plots above the red line indicate antagonism. (**A**) Scatter plot for lumefantrine and epirubicin combinations against D6 strain of *P. falciparum,* (**B**) Scatter plot for artemether and epirubicin combinations against D6 strain of *P. falciparum,* (**C**) Scatter plot for lumefantrine and epirubicin combination against 3D7 strain of *P. falciparum.* (**D**) Scatter plot for artemether and epirubicin combinations against 3D7 strain of *P. falciparum. (***E**) Scatter plot for lumefantrine and epirubicin combinations against W2 strain of *P. falciparum,* (**F**) Scatter plot for artemether and epirubicin combinations against W2 strain of *P. falciparum, (***G**) Scatter plot for lumefantrine and epirubicin combinations against clinical isolates, (**H**) Scatter plot for artemether and epirubicin combinations against clinical isolates.

**Figure 2:**
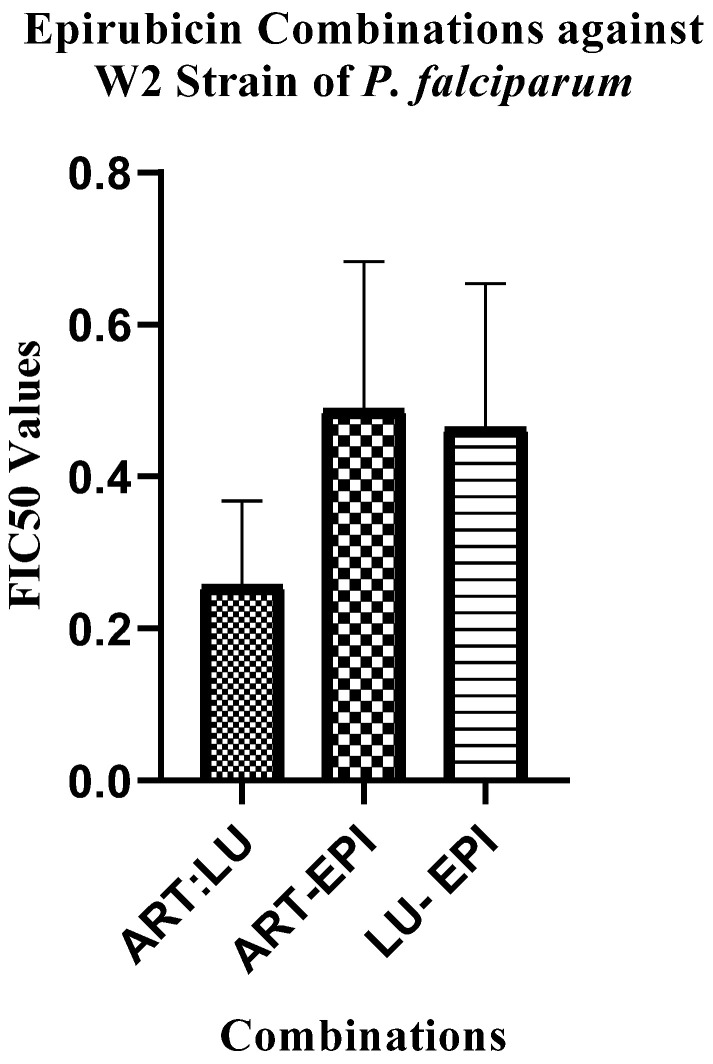
Epirubicin combinations compared with artemether (ART) and lumefantrine (LU) as a control against the W2 strain of *P. falciparum*.

All mean of mean sum FIC_50_ values for all separate combinations of epirubicin (EPI) with artemether (ART) and lumefantrine (LU) showed synergism (FIC_50_ < 0.7) across all fixed ratios when tested in vitro against different strains of *P. falciparum* and ex vivo against fresh field isolates (Table 1, Figure 1). Statistically significant differences were observed among the mean FIC_50_ values with a *p*-value of 0.0016, confirming the robustness of the synergistic interaction (Supplementary Figures S1–S4). Similar parasite suppression activities were observed when epirubicin combinations were compared with the antimalarial reference drug combinations (Figure 2).

The most notable synergistic effect was observed when LU was combined with EPI and tested against *P. falciparum* (3D7 strain) with a mean of mean sum FIC_50_ of 0.18 across all fixed ratios (Table 1, Figure 1C). Specifically, the drug–drug (LU:EPI) combination ratios of 1:1, 1:2, and 1:3 yielded mean sum FIC_50_ values of 0.058, 0.1, and 0.09, respectively, indicating the highest degree of synergism among the combinations tested (Table 1). However, some combinations showed additivity and antagonism as observed in scatter plots in Figure 1.

### 2.2. Antiplasmodial Activities of Artemether and Lumefantrine Each Combined with Pelitinib

**Figure 3:**
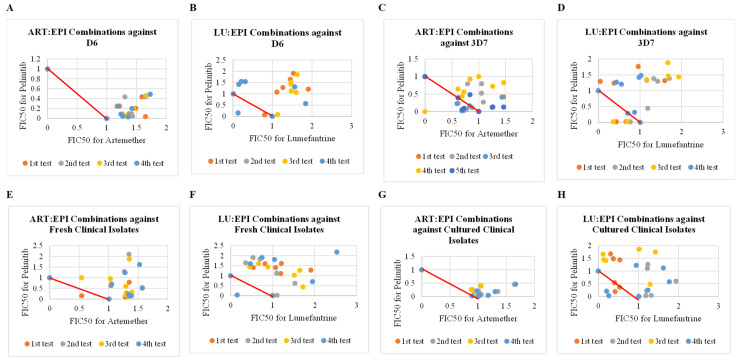
Interaction profiles of artemether and lumefantrine each combined with pelitinib against field isolates and cultured strains of *P. falciparum.* This figure presents a series of scatter plots illustrating the mean FIC_50_ values for combinations of artemether (ART) and lumefantrine (LU) with pelitinib (PEL). Each plot delineates the interactions against distinct strains of *P. falciparum* in vitro (D6 and 3D7) and ex vivo against clinical field isolates. Plots below the red line indicate synergism, plots along the red line indicate additivity, and plots above the red line indicate antagonism. (**A**) Scatter plot for lumefantrine and pelinitib combinations against D6 strain of *P. falciparum,* (**B**) Scatter plot for artemether and pelinitib combinations against D6 strain of *P. falciparum,* (**C**) Scatter plot for lumefantrine and pelinitib combination against 3D7 strain of *P. falciparum.* (**D**) Scatter plot for artemether and pelinitib combinations against 3D7 strain of *P. falciparum. (***E**) Scatter plot for lumefantrine and pelinitib combinations against W2 strain of *P. falciparum,* (**F**) Scatter plot for artemether and pelinitib combinations against W2 strain of *P. falciparum, (***G**) Scatter plot for lumefantrine and pelinitib combinations against clinical isolates, (**H**) Scatter plot for artemether and pelinitib combinations against clinical isolates.

In contrast to the synergistic outcomes observed with epirubicin combinations, the interactions of artemether (ART) and lumefantrine (LU) with pelitinib (PEL) predominantly resulted in additivity and antagonism. The mean of mean sum FIC_50_ values for these combinations consistently exceeded 1, indicative of additivity and antagonism interactions (Table 2, Figure 3). The highest antiplasmodial activity was displayed when artemether was combined with pelitinib and tested against the 3D7 strain of *P. falciparum* (Table 2). Most fixed combination ratios exhibited additivity and antagonism; however, synergism was observed at fixed ratios of 4:1 for both ART:PEL and LU:PEL, tested against the 3D7 strain of *P. falciparum* with FIC_50_ values of 0.75 and 0.8, respectively. Similarly, synergism was observed at fixed ratios of 3:1 for LU:PEL against D6 (FIC_50_ = 0.34) and ART:EPI against 3D7 (FIC_50_ = 0.83 and FIC_50_ = 0.91) strains of *P. falciparum* (Table 2). The antiplasmodial activities of the combinations were significantly different (*p* value = 0.0011) across different combinations and different strains of *P. falciparum* and clinical isolates (Figures S5–S8). Antiplasmodial activities of both fresh (ex vivo) and cultured (in vitro) were significantly different (*p* value = 0.0003) (Figures S7 and S8).

### 2.3. Antiplasmodial Activities of Artemether in Combination with Lumefantrine

**Figure 4:**
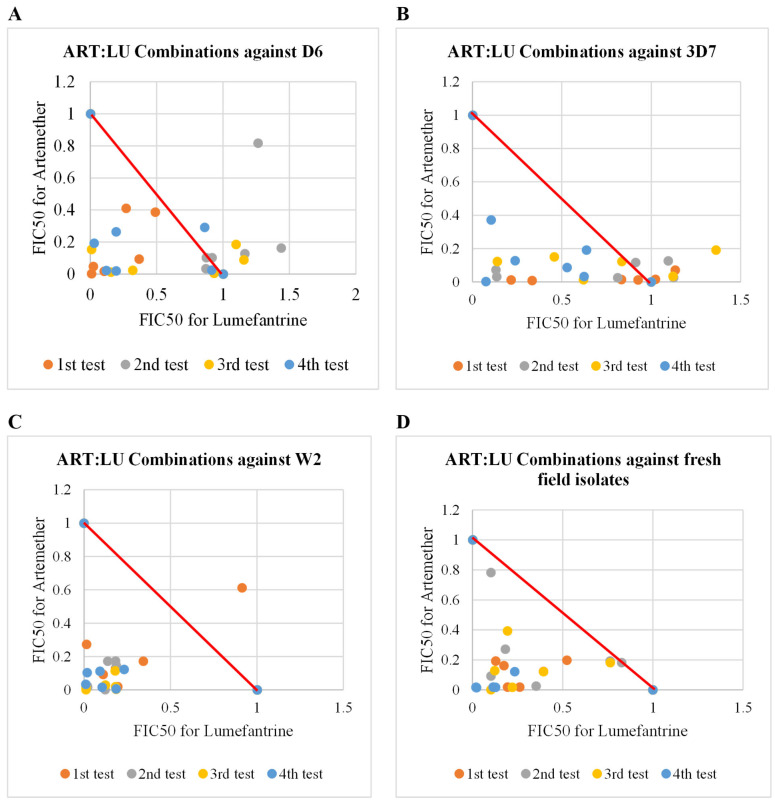
Scatter plots of artemether combined with lumefantrine against different strains of *P. falciparum* and field isolates. D6 and 3D7 = mefloquine-resistant; chloroquine-sensitive strains of *P. falciparum*; W2 = chloroquine-resistant, mefloquine-sensitive and artemisinin-sensitive Indochina strain of *P. falciparum*; Plots below the red line indicate synergism, plots along the red line indicate additivity, and plots above the red line indicate antagonism. (**A**) Artemether and lumefantrine combinations against D6 strain of *P. falciparum.* (**B**) Artemether and lumefantrine combinations against 3D7 strain of *P. falciparum.* (**C**) Artemether and lumefantrine combinations against W2 strain of *P. falciparum.* (**D**) Artemether and lumefantrine combinations against clinical isolates.

A combination of artemether (ART) and lumefantrine (LU) was used as a control for other drug combination assays. These assays revealed consistent synergism against various strains of *P. falciparum* and clinical isolates, with mean of the mean sum FIC_50_ values consistently below 0.8 (Table 3, Figure 4). The strongest synergistic effect was observed in combinations of ART with LU against the chloroquine-resistant, mefloquine-sensitive W2 strain of *P. falciparum* (Figure 4C). Across different fixed combination ratios, the mean of the mean sum FIC_50_ reached as low as 0.26, indicating a high degree of effectiveness (Table 3).

### 2.4. Antiplasmodial Activities of Reference Antimalarial Drugs

Our study evaluated the antimalarial efficacy of reference drugs. Potent antiplasmodial activities were displayed when epirubicin, artemether, lumefantrine, and mefloquine were tested against fresh clinical isolates and different strains of *P. falciparum* (W2, DD2, D6, 3D7 and F32 ART). DD2 is a chloroquine-resistant, mefloquine sensitive strain of *P. falciparum*; W2 is a chloroquine-resistant, mefloquine-sensitive, and artemisinin-sensitive Indochina strain of *P. falciparum*; D6 and 3D7 are mefloquine-resistant, chloroquine-sensitive strains of *P. falciparum*; and F32 ART is an artemisinin-resistant strain of *P. falciparum* (Table 4). Pelitinib showed good antiplasmodial activities (IC_50_ < 3) when tested across W2, DD2, D6, 3D7, and F32 ART strains of *P. falciparum* and excellent antiplasmodial activities against fresh clinical isolates (Table 4).

## 3. Discussion

Considering that drug therapy remains the main malaria management option, there is an urgency to cope with the ever-increasing occurrence of antimalarial drug-resistant parasites [5]. The emergence of resistant strains outpaces the introduction of new antimalarial drugs, primarily due to challenges of high cost, long time, and safety issues at different stages of drug discovery and development [21,23]. In addressing these hurdles, our study employed drug repurposing and combination therapy strategies, focusing on the antiplasmodial activities of pelitinib and epirubicin—both approved as anticancer agents. These drugs showed promising antiplasmodial activities in our previous study when tested independently [27]. Drug repurposing leverages existing drugs, potentially shortening the pathway to clinical application by bypassing early-phase testing and optimization. This approach utilizes compounds that are already approved or under investigation for new therapeutic purposes other than their original medical indications.

Recent advancements in computer-aided drug discovery (CADD) tools have shown potential to accelerate antimalarial drug discovery [22,39]. In silico methods such as drug repurposing have shown potential in overcoming antimalarial drug discovery challenges [40]. Particularly, in silico methods such as drug repurposing have been instrumental in navigating the complexities of drug development and have shown potential in overcoming antimalarial drug discovery [41].

Combination therapy is the most recommended first-line treatment of uncomplicated malaria [5], we combined PEL and EPI each with artemether and lumefantrine to explore their synergistic effects on antiplasmodial activity. The findings from the current study provide valuable insights into the feasibility of integrating novel drug discovery approaches with existing antimalarials to enhance the pipeline of increased drug candidates [42].

### 3.1. Combinations of Epirubicin with Artemether and Lumefantrine

Substantial synergism was observed (FIC_50_ < 0.7) in most combinations of epirubicin (EPI) with artemether (ART) and lumefantrine (LU) across different fixed drug–drug ratios when tested ex vivo against fresh field isolates and in vitro against different strains of *P. falciparum* (Table 1, Figure 1). The combinations against different strains of *P. falciparum* and clinical isolates showed FIC_50_ values of <0.7 that were significantly different (*p* value = 0.0016). The displayed synergism suggests the drugs used in combination potentiate each other towards increasing the overall observed antiplasmodial activities than when the drugs are tested alone. This portrays a potential use of epirubicin in antimalarial drug combinations.

Similarly, epirubicin displayed potent antiplasmodial activities when tested alone against fresh clinical isolates (ex vivo) and when tested against various strains of *P. falciparum* (in vitro) with IC_50_ values of <0.2. The observed high parasite clearance of epirubicin is similar to our previously reported study when the drug was tested alone against fresh clinical isolates (ex vivo) and various strains of *P. falciparum* (in vitro) with (IC_50_ < 0.4) [37]. Similar antiplasmodial activities of epirubicin were also reported in another study that utilized in silico chemogenomics approaches, where potent antiplasmodial activities were observed when the drug was tested against field isolates and in vitro using sensitive and multidrug-resistant strains of *P. falciparum* and *P. vivax* [43].

The mean of means of ART:LU combinations which were used as a control were comparable to ART:EPI and LU:EPI combinations (Table 1 and Table 3) when tested against D6 (Figure S1), 3D7 (Figure S2), W2 (Figure S3), and fresh clinical isolate (Figure S4) strains of *P. falciparum*, especially when tested against the W2 strain of *P. falciparum* that were significantly similar, with a *p* value of 0.0379 (Figure 2). The observed synergisms when ART and LU were each combined with epirubicin also show a potential inclusion of the drug to the existing list of antimalarial drug combinations. Furthermore, malaria is mostly treated using ACTs, and studies continue to report the occurrence of ACT-resistant malaria-causing parasites [8,44]. The current results of antiplasmodial activities of epirubicin combinations contribute information on the use of drug combinations which is likely to increase parasite clearance and reduce the chances of occurrence of resistant parasites [5] as two drugs contribute to the therapeutic activity.

Additional support for epirubicin as a potential antimalarial candidate comes from a past target similarity study that identified similarities between epirubicin and *P. falciparum* protein DNA topoisomerase II (NCBI accession number: XP_001348490.1) [36]. This protein is shown to be very druggable, as indicated by a druggability index of 0.8. Similarly, the *P. falciparum* protein DNA topoisomerase II has amino acids that are likely to play a critical role in the life of the parasite, with 61% CONSURF results reported by the same study [36].

Similar antimalarial studies have been reported in animal models. A study where approved drugs (lopinavir–ritonavir) for HIV/AIDS were combined with ART and LU showed complete suppression of parasite growth of *Plasmodium berghei* in a mouse model [45]. Equally, another study showed that vitamin C co-administration with artemether-lumefantrine led to increased parasite clearance over ART-LU alone when tested in mice infected with *P. berghei* [46]. These preclinical studies indicate the potential use of drug repurposing and combination assays as a potential alternative for new antimalarial drug discovery.

### 3.2. Combinations of Pelitinib with Artemether and Lumefantrine

Most combinations of pelinitib (PEL) with ART and LU showed additivity and antagonism across different fixed drug–drug combinations against fresh clinical isolates and *P. falciparum* strains (Table 2 and Figure 3). Pelitinib showed lower antiplasmodial activities compared to epirubicin when the drugs were tested alone and in combinations (Table 2 and Figure 3). The presence of additivity implies that the combined drugs do not enhance each other’s therapeutic effect, while antagonism indicates a reduction in therapeutic activity compared to when the drugs are administered singly [6,38]. The observed additivity and antagonism may be attributed to pelitinib’s pharmacokinetic properties or interactions at the molecular level that mitigate the combination effectiveness when combined with ART and LU. This is likely to have led to a lower or similar combined effect of the two drugs used in the combination with pelinitib than the effect of the individual drugs when used alone. The observed higher antiplasmodial activity of epirubicin than pelinitib could be due to a difference in their molecular targets or structure.

### 3.3. Antiplasmodial Activities of Epirubicin and Pelitinib When Tested Alone

For compounds/drugs that are tested individually, a 50% inhibition concentration (IC_50_) is considered as potent/excellent (<1 µM), good (1–20 µM), moderate (20–100 µM), low (100–200 µM), or inactive (>200 µM) [13]. When EPI was tested alone, good antiplasmodial activities (IC_50_ = 1–2.6 µM) were observed across different strains of *P. falciparum* with potent antiplasmodial activity (IC_50_ = 0.27 µM) observed when the drug was tested against ex vivo against fresh clinical isolates (Table 4). Similar antiplasmodial activities were displayed in previous studies [37,43], suggesting EPI’s potential as a standalone antimalarial therapy.

Generally, this study confirms the potential use of drug repurposing through drug combinations in drug discovery and development [40]. The observed synergism in most combinations and the potent-to-good antiplasmodial activities of the two drugs suggest that these drugs can be further be explored for their potential alternative use as antimalarials.

### 3.4. Safety of Epirubicin

Both epirubicin and pelitinib are used for the treatment of cancer [47]. Epirubicin belongs to the anthracycline family and is used for the management of breast cancer [46].

Treatment of breast cancer patients with anthracyclines and/or anti-HER2-targeted therapies are highly associated with cardiovascular toxicity [47]. This exposes breast cancer patients to cardiotoxicity [48]. Recent studies have reported potential alternatives to alleviate the epirubicin-induced cardiotoxicity. The use of cardioprotective drugs has shown a potential to reduce cardiotoxicity [49]. Another study suggests that the sequential administration of epirubicin with sialic acid modified as a carrier has the potential to reduce epirubicin-induced cardiotoxicity [50]. Correspondingly, tryptophan-derived 3-indolepropionic acid has been reported to have potential in alleviating EPI-induced cytotoxicity [46]. The use of propolis, rich in phenolics, has also shown activity against EPI-induced cardiotoxicity and nephrotoxicity [51]. These studies show potential alternatives in circumventing anticancer-drug-induced toxicities. Furthermore, these drug compounds can act as a scaffold for structure modification through medicinal chemistry to improve their safety, antimalarial efficacy, and dosage, especially for children under the age of 5 years and pregnant women.

### 3.5. Strengths and Limitations

#### 3.5.1. Strengths

The current study has notable strengths in antimalarial drug discovery. This study provides information on the potential combination of these drugs, especially epirubicin with artemether and lumefantrine as a potential antimalarial agent. Drug combination reduces the chances of the development of drug-resistant malaria-causing parasites (a major contributor to malaria prevalence) and increases overall therapeutic activity through synergism or potentiation.

Considering that drug discovery and development efforts are challenged by time, money, and safety issues, the current study offers knowledge that can act as a guide toward overcoming these challenges. This is because the suggested drugs are currently in use in the market and considered to be safe, therefore, overcoming safety challenges. Furthermore, the drug targets of these drugs are known and are similar to *P. falciparum* proteins. Therefore, the information reported in this study, coupled with the already known pharmacokinetics of the drugs, can be used by other researchers to explore safety and dosing regimens appropriate to children and pregnant women who bear the brunt of malaria.

#### 3.5.2. Limitations

A limitation of this study was the inability to test these drugs in animal models, both alone and in combination against malaria causing parasites. This could determine the potential transition of these drugs into clinical trials as antimalarials. The effects of drug–drug interactions on the drug metabolism and safety of the co-used drugs in combination were also not determined.

## 4. Materials and Methods

### 4.1. Test Drugs and Reference Antimalarial Drugs

The two test drugs epirubicin and pelitinib were provided by our collaborators at the University of Potsdam in Germany. Reference antimalarial drugs (artemether, lumefantrine, chloroquine, and mefloquine) were donated by World Wide Antimalarial Resistance Network (WWARN) External Quality Assurance Programme, Bangkok [52]. Chloroquine (CQ) and mefloquine (MQ) were used as controls when the drugs were tested alone and a combination of artemether (ART) and lumefantrine (LU) was used as control for combination assays. The drugs were dissolved and diluted as earlier reported [6] in readiness for the assay. The drugs were dissolved in 99.5% DMSO (Sigma-Aldrich, Waltham, MA, USA) followed by dilution in complete Roswell Park Memorial Institute (RPMI 1640) (Invitrogen Inc., Waltham, MA, USA) cell culture medium. This was performed to achieve serial dilutions of 0.488–250 ng/mL (ART, LU, and MQ), 1.953–1000 ng/mL (CQ), and 24.414–25,000 ng/mL (epirubicin and pelitinib) that were generated on a 96-well plate with DMSO ≤ 0.0875% [37].

### 4.2. Plasmodium falciparum Parasite Strains

The Biodefense and Emerging Infections Research Resources Repository (BEI Resources), National Institute of Allergy and Infectious Diseases (NIAID), provided the strains of *P. falciparum*; mefloquine-resistant, chloroquine-sensitive Sierra Leone I (D6); chloroquine-sensitive, mefloquine-sensitive (3D7); chloroquine-resistant, mefloquine-sensitive (DD2); chloroquine-resistant, mefloquine-sensitive, and artemisinin-sensitive Indochina (W2); and artemisinin-resistant (F32 ART) [15,53]. The antiplasmodial efficacy of the test drugs was determined when tested in vitro against these reference malaria parasite strains of *P. falciparum*.

### 4.3. Drug Combinations

The drug combinations were made as described in our previous combination study [6]. Artemether (ART) and lumefantrine (LU) were each dissolved in 99.5% DMSO at 1 mg/100 μL and then reduced to a concentration of 200 ng/mL using RPMI-1640 media, (Invitrogen Inc., Waltham, MA, USA). Similarly, epirubicin (EPI) and pelitinib (PEL) were each dissolved in 99.5% DMSO at 1 mg/100 μL and then reduced to a concentration of 25,000 ng/mL using RPMI-1640 media. A total of 10 mL for each drug (ART, LU, EPI, and PEL) was transferred to a pre-labeled 15 mL centrifuge tube and then vortexed to uniformity. Two drug samples to be combined (standard antimalarial drug and repurposed drug), for example, artemether and epirubicin, were pooled together at working concentrations of each drug sample by transferring volumes of each drug that are proportionate to fixed artemether/epirubicin ratios of 4:1, 3:1, 1:1, 1:2, 1:3 and 1:4, volume/volume. The combination ratios were selected based on our previous studies [6,38] and the maximum number of rows (8 rows) on a 96-well plate, 2 for drugs alone and 6 for combinations. Each combination ratio was then transferred to its own pre-labeled 15 mL centrifuge tube, for example, an artemether/epirubicin ratio of 4:1 to tube 1, then vortexed to uniformity and 3:1 to tube 2, then vortexed to uniformity; the same was achieved for all combination ratios [6]. The same process was repeated for all combinations (ART: EPI, LU: EPI, ART: PEL, LU: PEL, and a combination control of ART: LU).

Both PEL and EPI were tested alone and then each was combined with ART and LU at different (ART: PEL, LU: PEL, ART: EPI and LU: EPI) fixed drug–drug ratios of 1:0, 4:1, 3:1, 1:1, 1:2, 1:3, and 1:4 0:1 volume/volume as described in our previous studies [6,38]. Serial dilutions of each pair of the combination partners were performed on a 96-well plate.

A 300 μL working concentration of a reference drug (artemether or lumefantrine) to be combined with a test drug (EPI or PEL), for example, for a combination of artemether/epirubicin, 300 μL of artemether (1:0) was transferred to well 1A of row 1 and 300 μL of epirubicin (0:1) to well 1B of row 2 as separate test drugs of a 96-well plate. Similarly, 300 μL of each fixed-ratio combination (4:1, 3:1, 1:1, 1:2, 1:3, and 1:4) were all transferred to well 1C, all through to well 1H of the same 96-well plate. Once all wells in the first column (1A–1H) were filled with the test drugs, a 150 μL diluent of 10% complete medium with serum (CMS) was transferred to all other remaining wells on the plate. This meant that all wells in column 1 had 300 μL of test drugs, and all other wells had 150 μL of 10% CMS [6].

To initiate the serial dilutions, 150 μL of the test drugs in all wells of column 1 (wells 1A, 1B, 1C, 1D, 1E, 1F, 1G and 1H) were transferred to each well of the second column (wells 2A, 2B, 2C, 2D, 2E, 2F, 2G and 2H) using a an 8-way multichannel pipette, then gently mixed by aspirating and dispensing five times. Similarly, 150 μL in the wells of the second column (wells 2A, 2B, 2C, 2D, 2E, 2F, 2G and 2H) were transferred to the third column (wells 3A, 3B, 3C, 3D, 3E, 3F, 3G and 3H), then mixed smoothly by aspirating and dispensing five times. The same was repeated across all wells of the remaining columns of the entire plate. This implied a decrease in drug concentration by 2-fold across the columns, with the highest in the wells of column 1 and lowest in the wells in column 12. This resulted in a dilution range of 97.7–25,000 ng/mL for test drugs (EPI or PEL) and 0.2–200 ng/mL for artemether or lumefantrine. Likewise, there was a proportionate reduction in concentrations for fixed-ratio combinations. The final DMSO concentration was ≤0.0875%, as described by [54]. This same method was repeated for all combinations (LU: EPI, ART: PEL, LU: PEL), including the combination control of ART: LU [6].

The plates were kept frozen at −65 to −80 °C for use within 14 days. In readiness for the start of antiplasmodial assays, the plates were thawed at room temperature, and 12.5 μL was transferred to a new 96-well assay plate. The red blood cells (RBCs), *P. falciparum* parasites, and Roswell Park Memorial Institute (RPMI) media were then added. The antiplasmodial assay proceeded as explained below.

### 4.4. Antiplasmodial Activities

#### 4.4.1. Ex Vivo Antiplasmodial Assays

Both ex vivo and in vitro antiplasmodial assays were performed using the non-radioactive malaria SYBR Green I assay technique (Thermo Fisher Scientific, Hillsboro, OR, USA) [55] with modifications as described in our previous studies [18,56]. For ex vivo assays, fresh clinical isolates of blood samples were collected from consenting patients with malaria presenting themselves at Kombewa Sub-County hospital, which is 20 km from the central lab [57]. This enabled the immediate ex vivo antiplasmodial assay to be performed within 6 h of collection. Once the samples arrived in the lab, infection with falciparum malaria was confirmed and percentage parasitemia determined through microscopy [6,54]. Blood samples of 1% parasitemia mixed with media and red blood cells were added onto the pre-dosed 96-well plates of reference drugs and test samples to initiate the assay. These plates were then incubated under moisture and gas mixture of 5% CO_2_, 5% O_2_, and 90% N_2_ for 72 h at 37 °C. Then, the assay was terminated by adding lysis buffer containing SYBR Green I dye and incubated at room temperature in the dark for 24 h. The readings of the plates were then performed using a Tecan machine Genios^®^ Plus (Tecan Group Limited, Männedorf, Switzerland) which was set at an integration time of 40 µs, gain at 60, number of flashes at 10, and emission and excitation wavelengths of 535 nm and 485 nm, respectively [6].

#### 4.4.2. In Vitro Antiplasmodial Assays

Both clinical isolates of blood samples collected from consenting patients with malaria presenting themselves at a distant hospital from the central lab (Marigat Sub-County hospital) and laboratory-adapted reference strains of *P. falciparum* (3D7, D6, DD2, W2 F32-ART) were used for in vitro assays. Both the clinical isolates and strains of *P. falciparum* were maintained in continuous culture adapted in in vitro conditions to a 3–10% parasitemia. The parasitemia was lowered to 1%; then, media and red blood cells were added and transferred onto pre-dosed 96-well plates to initiate the assay of reference drugs and test samples [6]. The plates were incubated and terminated, and readings were performed as explained above in Section 4.4.1.

### 4.5. Data Analysis

For drugs tested singly, relative fluorescence units (RFUs) obtained from the readings of the test plates were used to determine a concentration that inhibits 50% of *P. falciparum* parasites (IC_50_) for each sample with appropriate dose–response curves using GraphPad Prism^®^ 8.0 Windows software (GraphPad Software, San Diego, CA, USA). Each assay was performed in at least triplicate and presented as mean ± standard deviation (mean IC_50_ ± SD).

For drug–drug combinations, the IC_50_s of the drugs were also determined when tested alone and in combination, then used to calculate a sum 50% fractional inhibition concentration (FIC_50_) of each combination using the formula shown below, as previously reported [6].

$$Sum\;FIC50=\frac{IC50\;of\;A\;in\;combination}{IC50\;of\;A\;alone}+\frac{IC50\;of\;B\;in\;combination}{IC50\;of\;B\;alone}$$

where **A** = epirubicin or pelitinib **B** = artemether or lumefantrine.

The mean sum of FIC_50_s were used to create scatter plots [58]. One-way analysis of variance (ANOVA) analysis was performed to determine whether there was a significant difference in mean IC_50_s and sum FIC_50_s of all samples tested against *P. falciparum* strains and field isolates.

## 5. Conclusions

Introducing a new drug into the market for clinical use takes a significant amount of time, involves high costs, and other drugs often fail to be approved due to safety issues. Our study addressed these challenges by leveraging drug repurposing and drug combination strategies of epirubicin and pelitinib. Since the drugs are already approved for different indications, there is a potential to expedite their approval for new antimalarial clinical use, thereby increasing lab–market rate transition in drug discovery and development.

Epirubicin combinations demonstrated synergism, enhancing its antimalarial efficacy when combined with ART and LU. Conversely, pelitinib combinations resulted in additivity and antagonism, underlining the need for further investigation of its use in antimalarial therapy. The results underscore the urgent need for new antimalarial agents to combat the increasing prevalence of resistant malaria parasites. In vivo studies using animal models are essential to confirm the observed ex vivo and in vitro antiplasmodial activities. Additionally, potential drug–drug interactions involving these agents should be thoroughly explored to ensure safety and efficacy in clinical applications. Preliminary compatibility or stability studies should be conducted to ensure that the combined drugs do not chemically interfere with one another, which could influence pharmacodynamic outcomes.

## Figures and Tables

**Table 1:** Synergistic activities of artemether and lumefantrine combinations with epirubicin against field isolates and strains of *P. falciparum.*

Drug Combinations ART/LU: EPI	Mean of Sum FIC_50_ ± STDEV							
	*P. falciparum* Strains (In Vitro)						Clinical Field Isolates	
	D6		3D7		W2		KOM (Ex Vivo)	
	ART-EPI	LU-EPI	ART-EPI	LU-EPI	ART-EPI	LU-EPI	ART-EPI	LU-EPI
4:1	0.63 ± 0.05	0.60 ± 0.23	0.65 ± 0.04	0.28 ± 0.09	0.82 ± 0.24	0.85 ± 0.73	1.04 ± 0.51	0.53 ± 0.35
3:1	0.46 ± 0.09	0.44 ± 0.18	0.43 ± 0.33	0.40 ± 0.31	0.59 ± 0.31	0.54 ± 0.33	1.14 ± 0.30	0.71 ± 0.57
1:1	0.82 ± 0.44	0.76 ± 0.57	0.53 ± 0.35	0.058 ± 0.05	0.52 ± 0.30	0.31 ± 0.21	0.67 ± 0.52	0.19 ± 0.07
1:2	0.68 ± 0.23	0.64 ± 0.44	0.48 ± 0.27	0.10 ± 0.05	0.24 ± 0.22	0.43 ± 0.34	0.26 ± 0.17	0.20 ± 0.13
1:3	0.21 ± 0.12	0.43 ± 0.21	0.59 ± 0.42	0.09 ± 0.05	0.48 ± 0.36	0.33 ± 0.25	0.44 ± 0.32	0.18 ± 0.12
1:4	0.27 ± 0.19	0.39 ± 0.24	0.51 ± 0.34	0.13 ± 0.14	0.28 ± 0.13	0.33 ± 0.20	0.39 ± 0.27	0.28 ± 0.16
Mean *	0.51 ± 0.19	0.54 ± 0.31	0.53 ± 0.36	0.18 ± 0.11	0.49 ± 0.29	0.46 ± 0.34	0.65 ± 0.42	0.35 ± 0.28

*p* value = 0.0016; number of replicates = at least 4; ART = artemether; LU = lumefantrine; EPI = epirubicin; D6 and 3D7 = mefloquine-resistant, chloroquine-sensitive strains of *P. falciparum*; W2 = chloroquine-resistant, mefloquine sensitive strain of *P. falciparum*; Sum FIC_50_ = synergism < 1; additivity 1; antagonism > 1; KOM = Kombewa Sub-County hospital; Mean * = mean of mean FIC_50_s across all fixed ratios; STDEV = standard deviation.

**Table 2:** Mean sum FIC_50_ values for artemether and lumefantrine combinations with pelitinib against field isolates and D6 and 3D7 strains of *P. falciparum.*

Drug Combinations ART/LU: PEL	Mean of Sum FIC_50_ ± STDEV							
	*P. falciparum* Strains (In Vitro)				Clinical Field Isolates			
	D6		3D7		KOM (Ex Vivo)		MGT (In Vitro)	
	ART-PEL	LU-PEL	ART-PEL	LU-PEL	ART-PEL	LU-PEL	ART-PEL	LU-PEL
4:1	1.48 ± 0.13	1.95 ± 0.92	0.75 ± 0.06	0.8 ± 0.12	1.48 ± 0.06	1.57 ± 1.00	1.03 ± 0.07	1.11 ± 1.08
3:1	1.43 ± 0.18	0.34 ± 0.12	0.83 ± 0.11	0.91 ± 0.52	1.58 ± 0.08	2.72 ± 0.38	1.14 ± 0.08	1.62 ± 0.66
1:1	1.41 ± 0.07	2.69 ± 0.31	1.36 ± 0.01	2.33 ± 0.43	1.91 ± 0.21	2.93 ± 1.02	1.12 ± 0.01	1.99 ± 0.58
1:2	1.65 ± 0.03	2.13 ± 0.33	1.19 ± 0.27	2.94 ± 0.43	1.78 ± 0.12	2.55 ± 0.19	1.31 ± 0.2	2.28 ± 0.44
1:3	1.43 ± 0.02	2.31 ± 0.59	1.86 ± 0.01	2.24 ± 0.73	2.6 ± 0.39	2.44 ± 0.14	1.17 ± 0.03	1.13 ± 0.61
1:4	2.12 ± 0.07	2.29 ± 0.79	1.08 ± 0.35	2.62 ± 0.61	2.2 ± 1.13	1.97 ± 0.07	1.78 ± 0.33	2.12 ± 0.23
Mean *	1.58 ± 0.08	1.95 ± 0.51	1.18 ± 0.14	1.97 ± 0.47	1.93 ± 0.33	2.36 ± 0.47	1.26 ± 0.12	1.71 ± 0.06

*p* value = 0.0011; number of replicates = 4; ART = artemether; LU = lumefantrine; PEL = pelitinib; D6 and 3D7 = mefloquine-resistant, chloroquine-sensitive strains of *P. falciparum*; Sum FIC_50_ = synergism < 1; additivity 1; antagonism > 1; KOM = Kombewa Sub-County hospital; MGT = Marigat Sub-County hospital; Mean * = mean of mean sum FIC_50_s across all fixed ratios; STDEV = standard deviation.

**Table 3:** Mean sum FIC_50_ values of artemether and lumefantrine combinations against field isolates and strains of *P. falciparum* (D6, 3D7, and W2).

Drug Combinations ART:LU	Mean of Sum FIC_50_ ± STDEV			
	*P. falciparum* Strains (In Vitro)			Clinical Field Isolates
	D6	3D7	W2	KOM (Ex Vivo)
	ART:LU	ART:LU	ART:LU	ART:LU
4:1	0.94 ± 0.44	1.07 ± 0.30	0.26 ± 0.06	0.66 ± 0.32
3:1	1.01 ± 0.23	0.92 ± 0.34	0.25 ± 0.08	0.55 ± 0.29
1:1	1.06 ± 0.67	0.91 ± 0.26	0.18 ± 0.07	0.26 ± 0.15
1:2	0.62 ± 0.50	0.83 ± 0.23	0.43 ± 0.33	0.37 ± 0.30
1:3	0.37 ± 0.25	0.31 ± 0.21	0.34 ± 0.11	0.27 ± 0.27
1:4	0.34 ± 0.29	0.32 ± 0.18	0.08 ± 0.06	0.32 ± 0.17
Mean *	0.72 ± 0.43	0.72 ± 0.25	0.26 ± 0.17	0.40 ± 0.25

*p* value = 0.0012; number of replicates = 4; ART = artemether; LU = lumefantrine; D6 and 3D7 = mefloquine-resistant, chloroquine-sensitive strains of *P. falciparum*; W2 = chloroquine-resistant, mefloquine-sensitive, and artemisinin-sensitive Indochina strain of *P. falciparum*; Sum FIC_50_ = synergism < 1; additivity 1; antagonism > 1; KOM = Kombewa Sub-County hospital; MGT = Marigat Sub-County hospital; Mean* = mean of mean sum FIC_50_s across all fixed ratios; STDEV = standard deviation.

**Table 4:** Mean IC_50_s values of drugs when tested alone.

Drugs	Mean IC_50_ ± STDEV (µM)					
	*P. falciparum* Strains					Field Isolates (Ex Vivo)
	W2	DD2	D6	3D7	F32 ART	KOM
Epirubicin	0.08 ± 0.01	0.12 ± 0.04	0.17 ± 0.01	0.15 ± 0.02	0.18 ± 0.03	0.08 ± 0.01
Pelitinib	1.21 ± 0.09	1.36 ± 0.29	2.62 ± 0.39	1.48 ± 0.31	1.41 ± 0.07	0.27 ± 0.01
Artemether	0.0046 ± 0.0016	0.011 ± 0.006	0.0032 ± 0.001	0.0052 ± 0.002	0.004 ± 0.001	0.008 ± 0.003
Lumefantrine	0.061 ± 0.042	0.026 ± 0.0053	0.049 ± 0.003	0.072 ± 0.005	0.21 ± 0.06	0.046 ± 0.007
Mefloquine	0.0043 ± 0.0012	0.066 ± 0.001	0.0091 ± 0.003	0.0042 ± 0.002	0.038 ± 0.003	0.0058 ± 0.002
Chloroquine	0.12 ± 0.02	0.263 ± 0.04	1.37 ± 0.04	0.095 ± 0.03	0.015 ± 0.005	0.18 ± 0.05

*p* value = 0.6779; number of replicates: 4; STDEV = standard deviation; D6 and 3D7 = mefloquine-resistant; chloroquine-sensitive strains of *P. falciparum*; DD2 = chloroquine-resistant, mefloquine-sensitive strain of *P. falciparum*; W2 = chloroquine-resistant, mefloquine-sensitive and artemisinin-sensitive Indochina strain of *P. falciparum*; F32 ART = artemisinin-resistant strain of *P. falciparum*; KOM = Kombewa Sub-County hospital; STDEV = standard deviation.

## Data Availability

The original contributions presented in this study are included in the article/Supplementary Material. Further inquiries can be directed to the corresponding author.

## Supplementary Materials

The following supporting information can be downloaded at: https://www.mdpi.com/article/10.3390/ph18070956/s1, Figure S1: Artemether (ART) and lumefantrine (LU) each combined with epirubicin (EPI) compared with ART combined with LU against P. falciparum (D6 strain); Figure S2: Artemether (ART) and lumefantrine (LU) each combined with epirubicin (EPI) compared with ART combined with LU against P. falciparum (3D7 strain); Figure S3: Artemether (ART) and lumefantrine (LU) each combined with epirubicin (EPI) compared with ART combined with LU against P. falciparum (3D7 strain); Figure S4: Artemether (ART) and lumefantrine (LU) each combined with epirubicin (EPI) compared with ART combined with LU against fresh field isolates (ex vivo); Figure S5: Artemether (ART) and lumefantrine (LU) each combined with pelinitib (PEL compared with ART combined with LU against P. falciparum (D6 strain); Figure S6: Artemether (ART) and lumefantrine (LU) each combined with pelinitib (PEL) compared with ART combined with LU against P. falciparum (3D7 strain); Figure S7: Artemether (ART) and lumefantrine (LU) each combined with epirubicin (EPI) compared with ART combined with LU against fresh field isolates (ex vivo); Figure S8: Artemether (ART) and lumefantrine (LU) each combined with epirubicin (EPI) compared with ART combined with LU against field isolates (in vitro).

## List of Abbreviations

ACTs: artemisinin-based combination therapies; AL: artemether–lumefantrine; ANOVA: one-way analysis of variance; ART: artemether; AS-AQ: artesunate–amodiaquine; CMS: complete medium with serum; CQ: Chloroquine; DHA-PPQ: dihydroartemisinin-piperaquine; DMSO: dimethylsulphoxide; EPI: epirubicin; FIC_50_: fifty per cent fractional inhibition concentration; IC_50_: fifty per cent parasite inhibition concentration; LU: lumefantrine; MQ: mefloquine; NCBI: National Center for Biotechnology Information; NIAID: National Institute of Allergy and Infectious Diseases; PEL: pelitinib; RFUs: relative fluorescence units; WHO: World Health Organization; WWARN: WorldWide Antimalarial Resistance Network.
