# Supplementary material for: Antimalarial Drug Repurposing of Epirubicin and Pelitinib in Combination with Artemether and Lumefantrine
Source: Pharmaceuticals (Basel). 2025 Jun 25;18(7):956. doi: 10.3390/ph18070956 (PMC12298365; doi:10.3390/ph18070956)

Supplementary Materials

Graphs of Mean Sum FIC50s of drug combinations

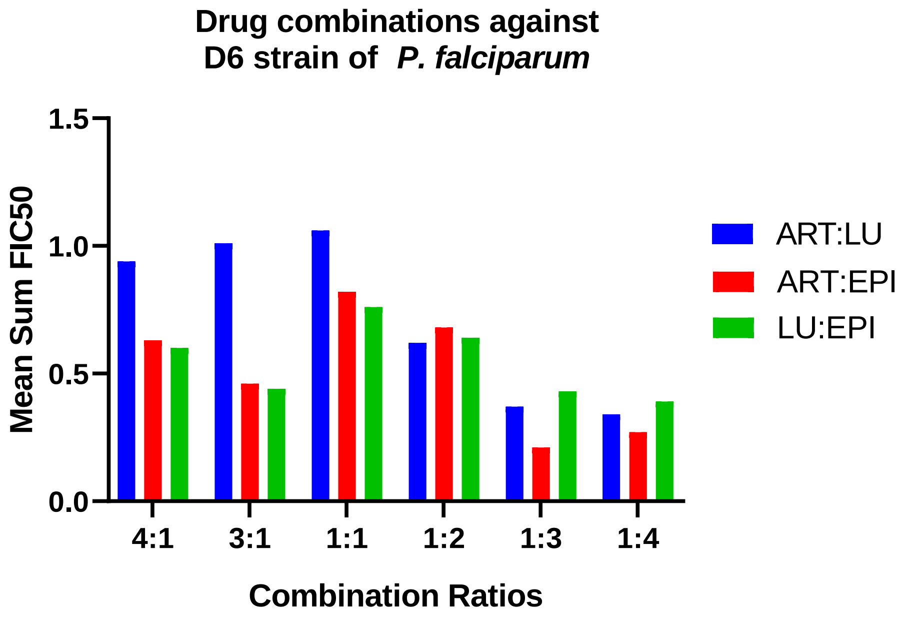

Figure S1: Artemether (ART) and lumefantrine (LU) each combined with epirubicin (EPI) compared with ART combined with LU against *P. falciparum* (D6 strain)

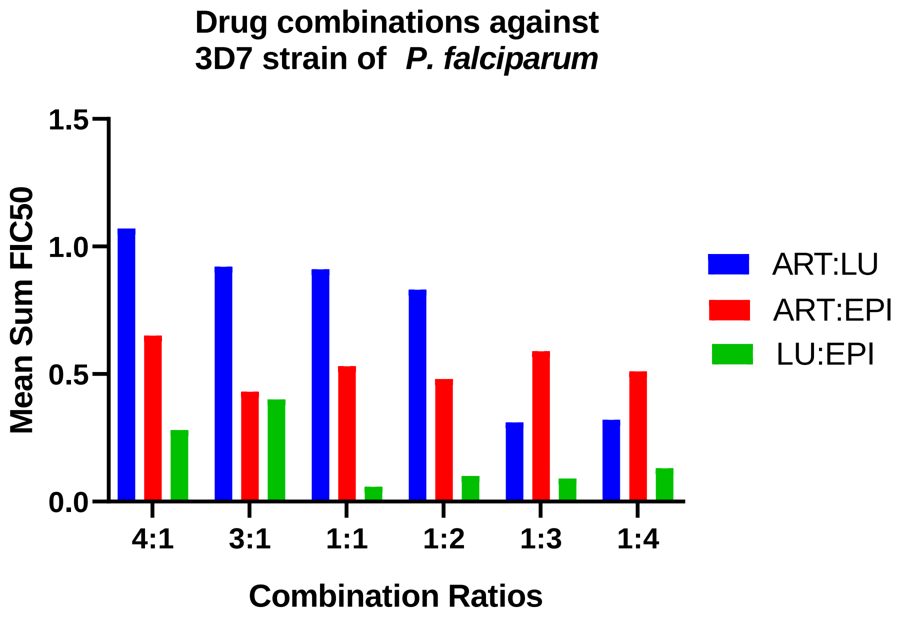

Figure S2: Artemether (ART) and lumefantrine (LU) each combined with epirubicin (EPI) compared with ART combined with LU against *P. falciparum* (3D7 strain)

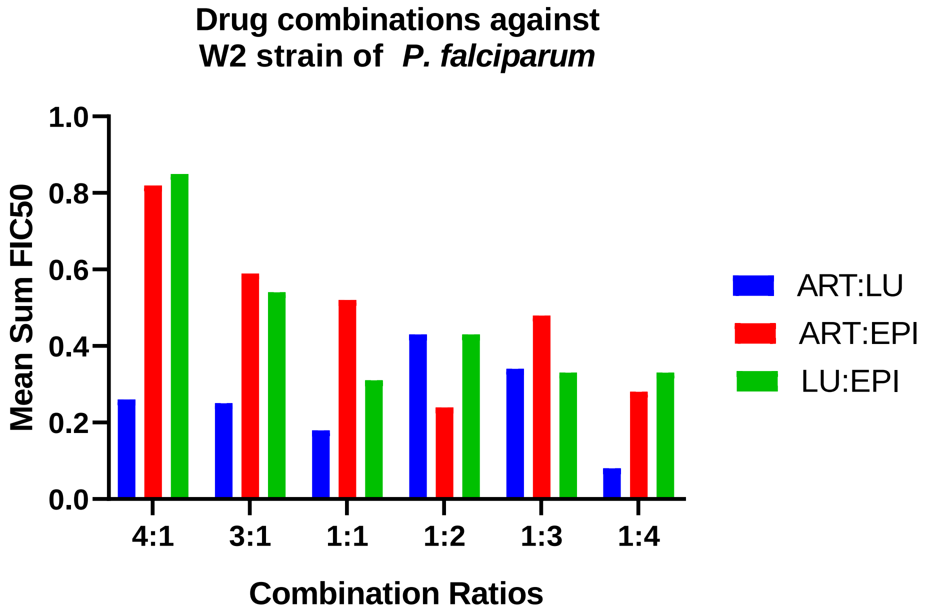

Figure S3: Artemether (ART) and lumefantrine (LU) each combined with epirubicin (EPI) compared with ART combined with LU against *P. falciparum* (3D7 strain)

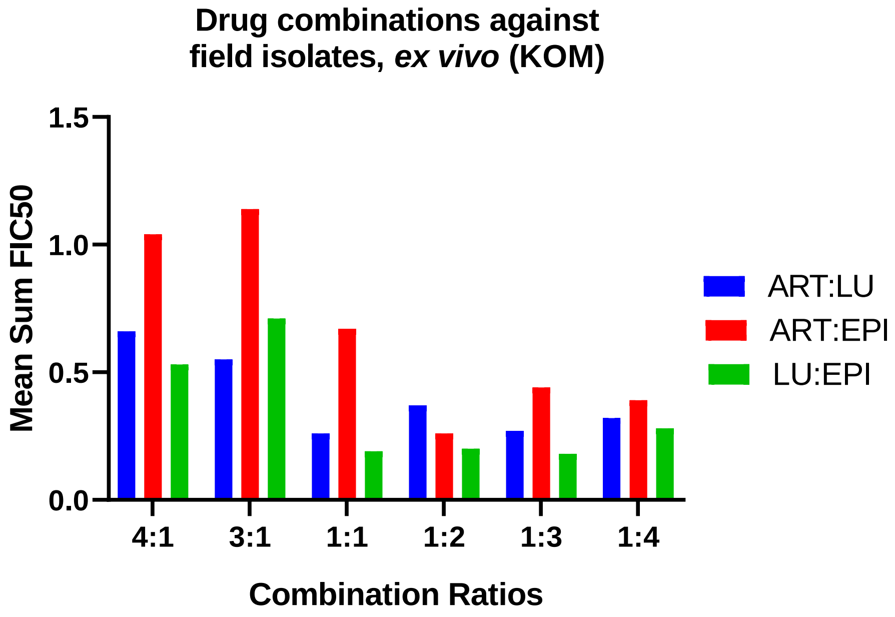

Figure S4: Artemether (ART) and lumefantrine (LU) each combined with epirubicin (EPI) compared with ART combined with LU against fresh field isolates (*ex vivo*)

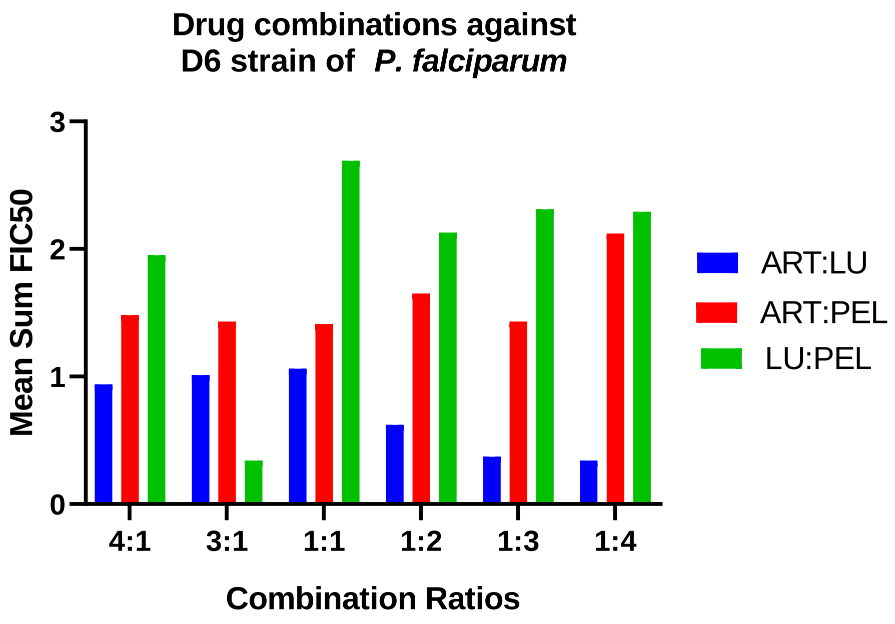

Figure S5: Artemether (ART) and lumefantrine (LU) each combined with pelinitib (PEL compared with ART combined with LU against *P. falciparum* (D6 strain)

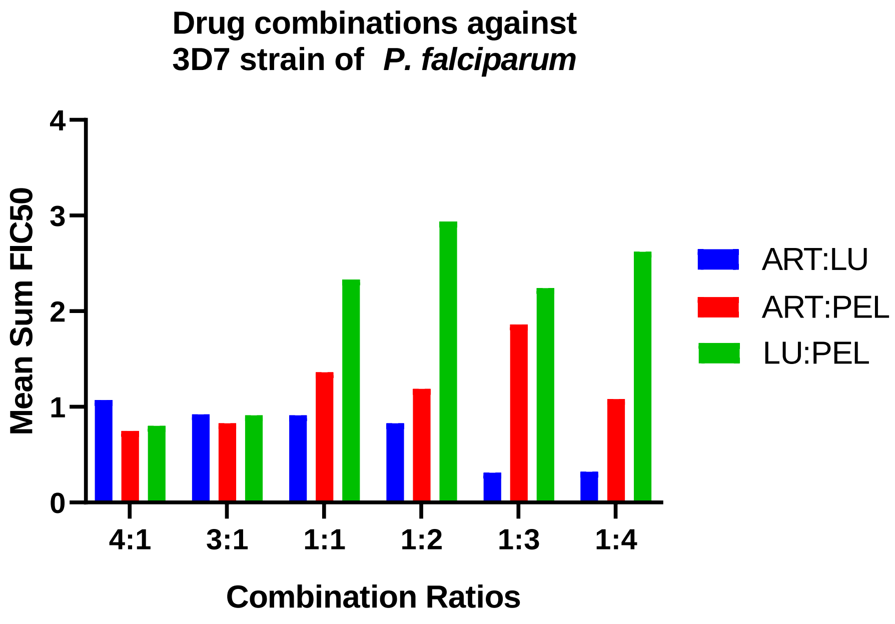

Figure S6: Artemether (ART) and lumefantrine (LU) each combined with pelinitib (PEL) compared with ART combined with LU against *P. falciparum* (3D7 strain)

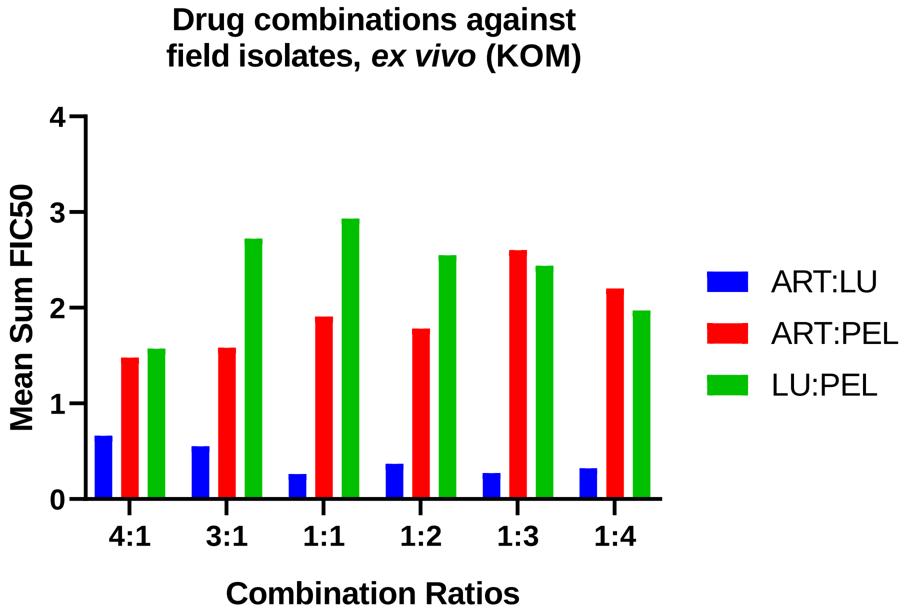

Figure S7: Artemether (ART) and lumefantrine (LU) each combined with epirubicin (EPI) compared with ART combined with LU against fresh field isolates (*ex vivo*)

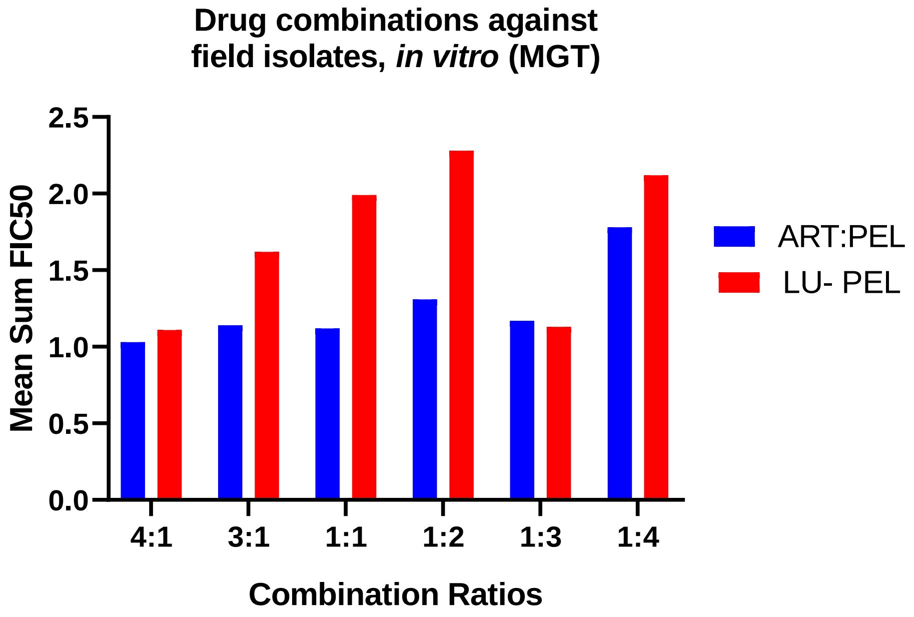

Figure S8: Artemether (ART) and lumefantrine (LU) each combined with epirubicin (EPI) compared with ART combined with LU against field isolates (*in vitro*)

Selected Antiplasmodial Activity Dose-Response Curves of Pelinitib

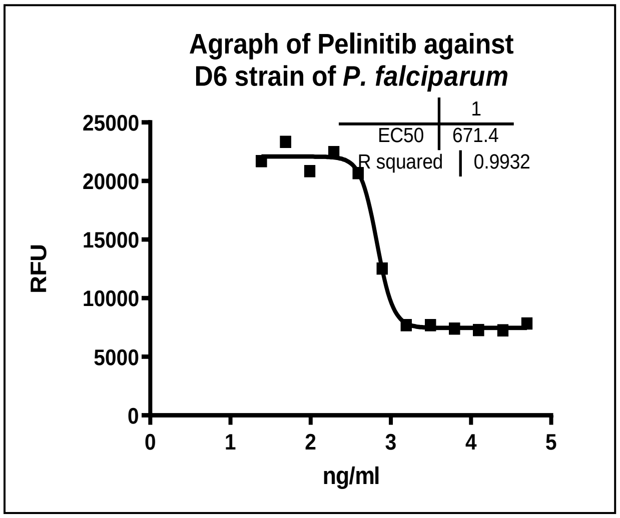

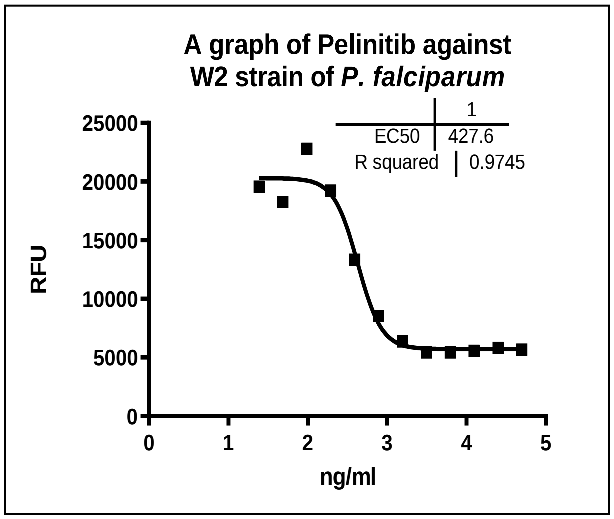

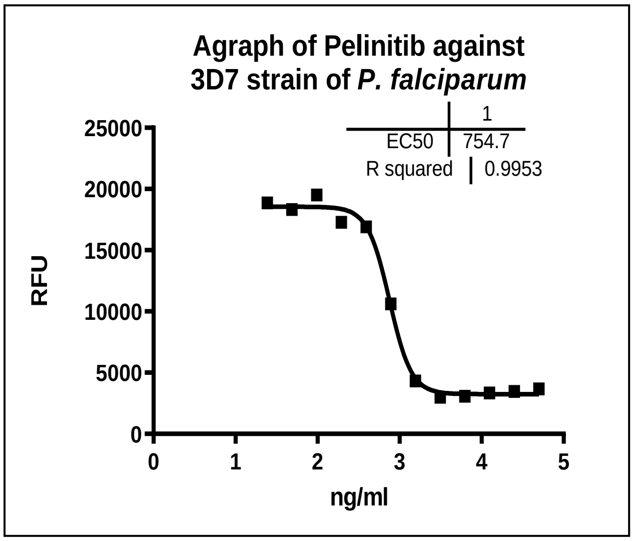

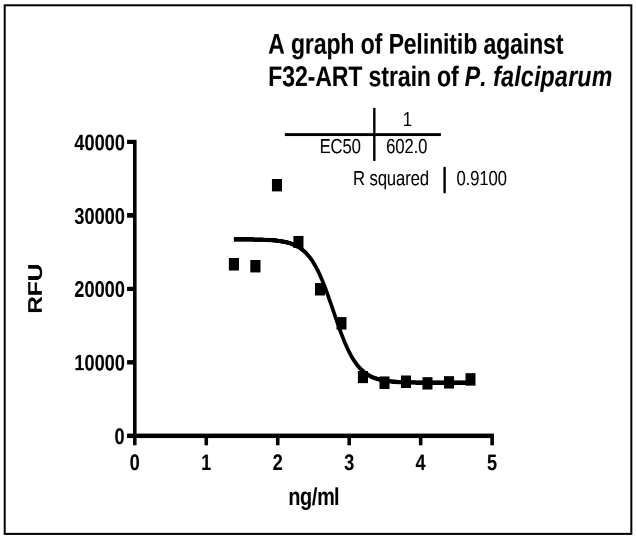

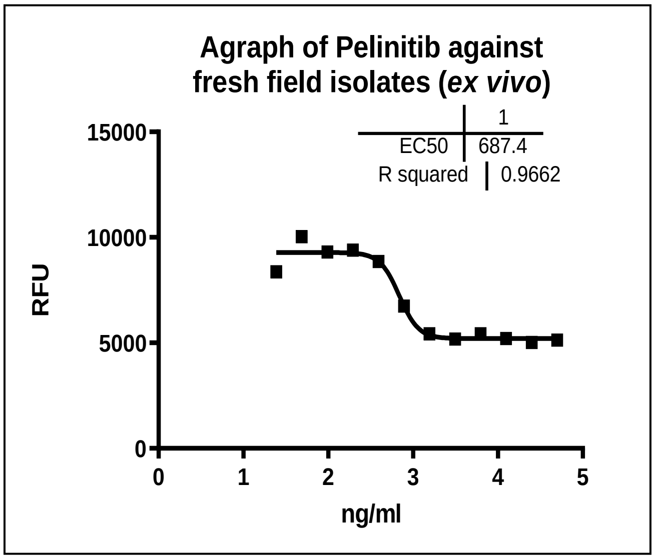

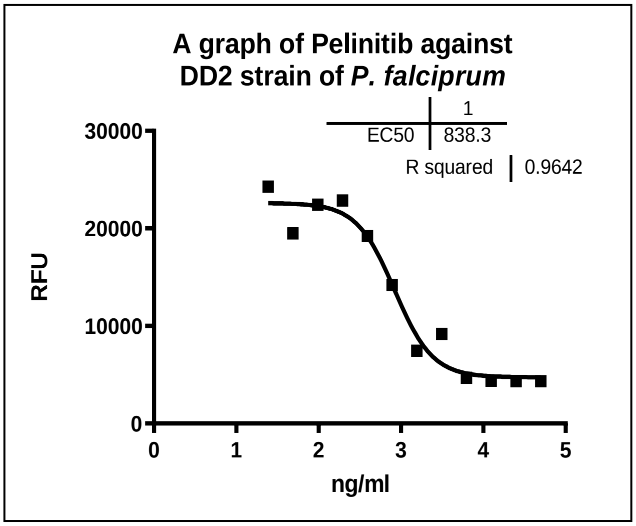

Selected Antiplasmodial Activity Dose-Response Curves of Epirubicin

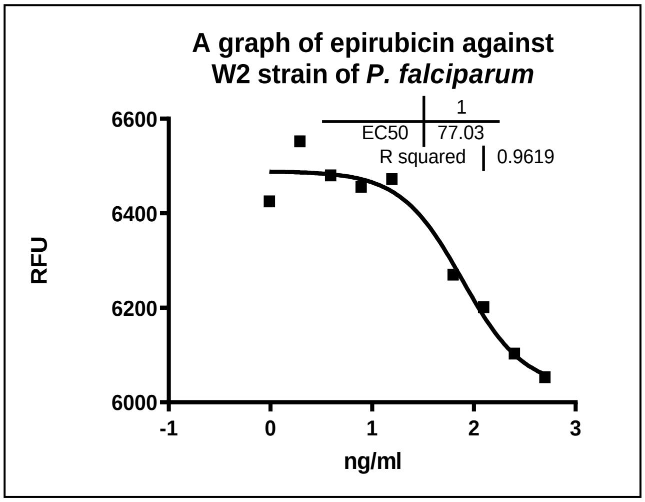

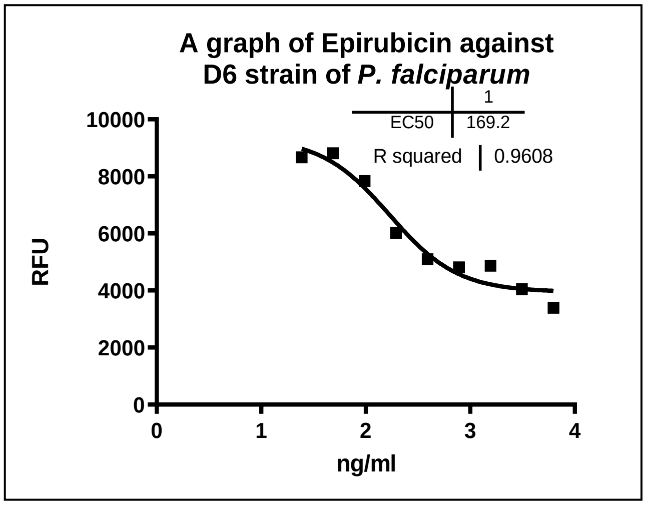

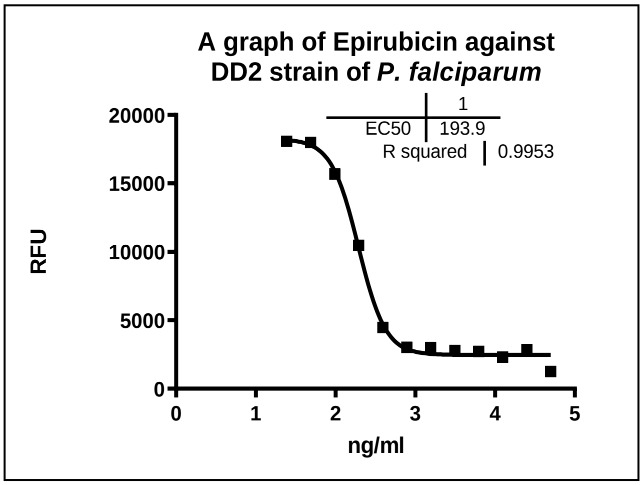

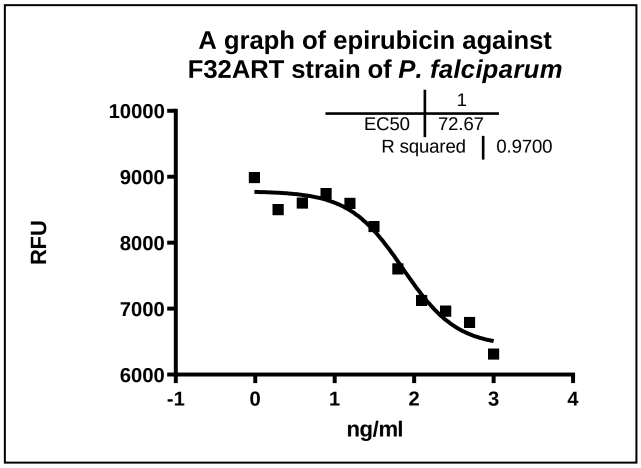

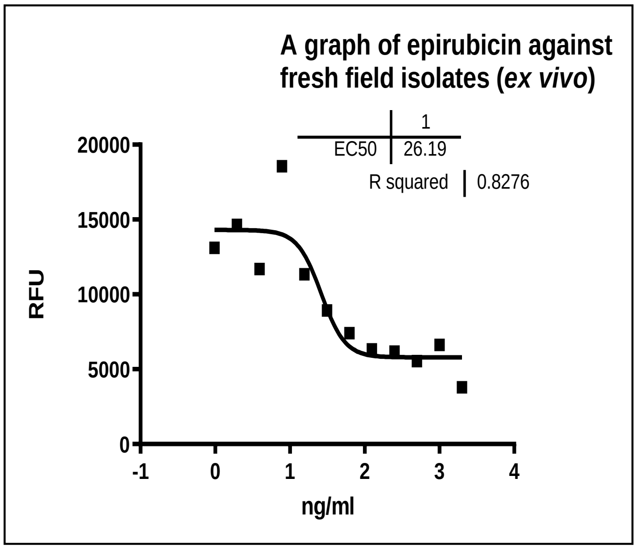

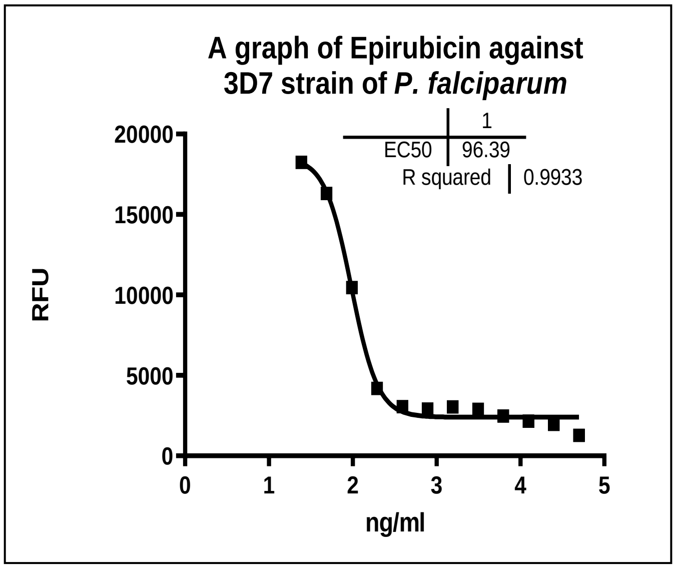

Supplement: Supplementary file 1 [file pharmaceuticals-18-00956-s001.zip › pharmaceuticals-3642725-supplementary.pdf]
